# Molecular Insights into Central Core Disease: Proteomic Signatures and Potential Therapeutic Biomarkers in RYR1 I4895T Mice

**DOI:** 10.3390/ijms262311451

**Published:** 2025-11-26

**Authors:** Lorenza Vantaggiato, Enxhi Shaba, Federica Fiore, Daniela Rossi, Vincenzo Sorrentino, Luca Bini, Claudia Landi

**Affiliations:** 1 Functional Proteomics Lab., Department of Life Sciences, University of Siena, 53100 Siena, Italy; lorenz.vantaggiato2@unisi.it (L.V.); enxhi.shaba@unisi.it (E.S.); claudia.landi@unisi.it (C.L.); 2 Department of Molecular and Developmental Medicine, University of Siena, 53100 Siena, Italy; federica.fiore@student.unisi.it (F.F.); daniela.rossi@unisi.it (D.R.); vincenzo.sorrentino@unisi.it (V.S.); 3 Program of Molecular Diagnosis of Rare Genetic Diseases, Azienda Ospedaliera Universitaria Senese, 53100 Siena, Italy

**Keywords:** Central Core Disease, RYR1, glycolysis, oxidative phosphorylation, oxidative stress, PGC1α

## Abstract

Central Core Disease (CCD) is a congenital myopathy predominantly caused by mutations in the gene encoding ryanodine receptor type-1 (RYR1), the intracellular Ca^2+^ release channel embedded in the skeletal muscle sarcoplasmic reticulum membrane. The I4898T mutation represents one of the most common RYR1 mutations associated with CCD. Unfortunately, there are no approved therapies for CCD or for other myopathies caused by mutations in this gene. This study aims to perform a top-down differential proteomic analysis on soleus muscle samples from wild-type mice (WT) and heterozygous knock-in mice carrying the I4895T (IT) mutation in RyR1, to investigate the pathogenic mechanisms and molecular pathways involved in this myopathy and to shed light on new potential biomarkers useful for future therapies. Proteomic analysis revealed 50 dysregulated protein species, and multivariate analysis showed that IT mice exhibit a distinct proteomic signature compared to WT mice, characterized by alterations in proteins associated with contractile and structural dysfunction, metabolism, and stress response. In particular, a significant increase in myosin fragments was observed in IT mice, likely due to muscle breakdown. In contrast, myotilin was downregulated, suggesting a weakening of the muscle cytoskeletal structure. There was a notable downregulation of proteins involved in glycolysis and the TCA cycle; conversely, there was an increase in proteins related to anaerobic glycolysis, suggesting a shift from aerobic to anaerobic glycolysis. Furthermore, proteins involved in fatty acid beta-oxidation and oxidative phosphorylation were also found to be upregulated in IT mice, indicating an attempt by the muscle to maximize energy production. Finally, we found a significant decrease in PGC1α, which could serve as potential therapy target and biomarker in CCD.

## 1. Introduction

The *RYR1* gene encodes the ryanodine receptor 1 (RYR1), a large tetrameric intracellular Ca^2+^ release channel of approximately 2.2 MDa, located on the sarcoplasmic reticulum (SR) of skeletal muscle. RYR1 channels play a pivotal role in excitation–contraction (EC) coupling, the process by which an action potential is transduced into Ca^2+^ release from the SR, resulting in physiological activation of muscle contraction [1,2,3].

From a genetic perspective, dominant mutations in *RYR1* are the principal cause of malignant hyperthermia (MH), a potentially lethal reaction triggered by certain anesthetics or, less commonly, by extreme environmental heat and/or intense physical exertion [3,4,5,6]. In addition to causing MH, *RYR1* mutations also cause congenital non-dystrophic muscle myopathies [1,2,3,4,7,8,9,10,11,12,13,14,15,16]. These myopathies, which may show dominant or recessive inheritance patterns, are collectively known as *RYR1*-related myopathies (RYR1-RM) [3,4]. Clinical presentation is highly variable, ranging from mild, non-progressive muscle weakness to severe forms requiring walking assistance or wheelchair use [4,8,13,14]. Feeding difficulties and respiratory involvement may also occur, potentially leading to the need for mechanical ventilation and reduced lifespan expectancy [7,10,11]. Central Core Disease (CCD) is the most frequent congenital myopathy and, in approximately 90% of cases, is caused by *RYR1* mutations [12]. At the histopathological level, muscle biopsies from individuals with CCD show distinctive amorphous central regions, or cores, characterized by a conspicuous absence or depletion of mitochondrial and oxidative enzyme activity, increased internal and central nuclei, Z-line streaming, sarcomeric disorganization, and a predominance of type I muscle fibers [8,9]. Although it is widely accepted that all *RYR1* mutations disrupt Ca^2+^ homeostasis, the mechanisms underlying muscle damage and hypotrophy remain poorly understood [7]. Currently, no specific treatment is available for CCD, and patient management is primarily supportive, employing a multidisciplinary approach such as daily physiotherapy [15].

The I4898T mutation in *RYR1* is one of the most common mutations associated with CCD [4,9,11,12]. This I4898T mutation, localized in the pore region of the RYR1 channel, causes EC coupling disruption by abolishing or severely reducing Ca^2+^ release channel conductance [17,18,19]. Homozygous mice carrying the *Ryr1* I4895T mutations (equivalent to I4898T in humans) show a severe phenotype, with paralysis at birth, inability to breathe, perinatal lethality, and delayed development of skeletal and cardiovascular systems [18,20]. Heterozygous *Ryr1* I4895T mice (from now on referred to as IT mice) exhibit a mild myopathy characterized by diminished muscle performance and reduced Ca^2+^ transients amplitude in skeletal muscle fibers [17,19,21]. However, it is important to note that the phenotype of IT mice is relatively mild when compared with the clinical manifestations observed in humans [22]. Studies by Lee et al., 2017 [20] suggested that a persistent increase in ER stress/Unfolded Protein Response (UPR), increased in mitochondrial Ca^2+^ uptake, reactive oxygen species (ROSs) production, and induction of apoptosis may represent possible pathogenic mechanisms for CCD associated with the Ryr1 I4895T mutation.

To further investigate the pathogenic mechanisms and molecular pathways involved in this myopathy, and to shed light on new potential biomarkers useful for future therapeutic strategies or for delaying disease progression, we performed a top-down differential proteomic analysis on soleus muscles from wild-type mice and heterozygous IT knock-in mice.

## 2. Results and Discussion

The clinical course for the CCD patients is static or slowly progressive. While cardiac complications are uncommon, respiratory muscle involvement often has an insidious onset. Consequently, serial pulmonary function tests are necessary for lung function monitoring [11]. Furthermore, pediatric patients require surveillance for the development of scoliosis and other skeletal deformities [23]. Unfortunately, despite a medical necessity, there are currently no approved therapies for CCD [15]. Therapies currently under development include drugs targeting the RYR1 signaling axis, either by mitigating the cellular pathology arising from oxidative/nitrosative stress and subsequent RYR1 post-translational modification, or by direct modulating RYR1 activity or indirectly influencing its function through interaction with regulatory proteins. A top-down proteomic analysis can be valuable for enhancing knowledge of alterations in protein abundance or function, including different proteoforms, as well as changes in molecular mechanisms caused by pathology, aiding in the identification of novel therapeutic biomarkers for patient management.

### 2.1. WT and IT Mice Muscles Present a Differential Protein Pattern

**Figure 1:**
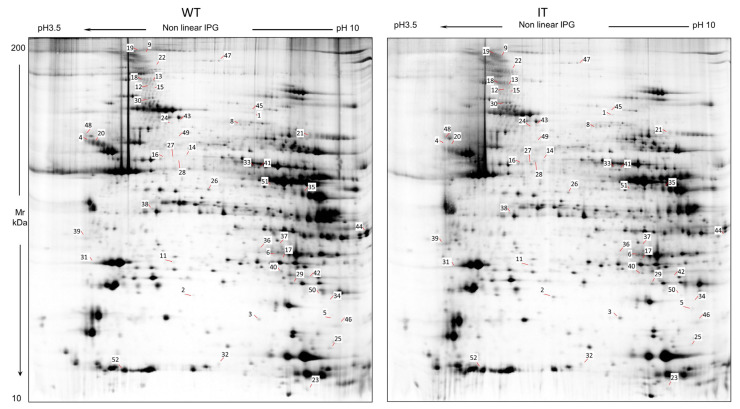
WT and IT soleus 2D gel master maps reporting differential abundant spots (*p* ≤ 0.05) with a fold-change ratio ≥ 1.5.

To understand how mutations in the RYR1 gene affect cellular mechanisms in skeletal muscle tissues, we applied a top-down differential proteomic analysis between soleus samples from wild-type mice (WT) and heterozygous mice knock-in for RYR1 I4895T (IT). The use of a top-down proteomics approach has the advantage of considering both proteins and protein species, or proteoforms resulting from their post-translational modifications, thereby overcoming the dilemma of their quantification [24]. Based on our previous results and to increase the number of separated proteins, we used two different protein extraction methods [25]. About 2500 protein spots per gel were detected using image analysis software (Melanie 9.0), statistical analysis highlighted 50 differentially abundant spots (Table 1, Figure 1 and Figure S1), of which 43 were successfully identified using MALDI-ToF.

**Figure 2:**
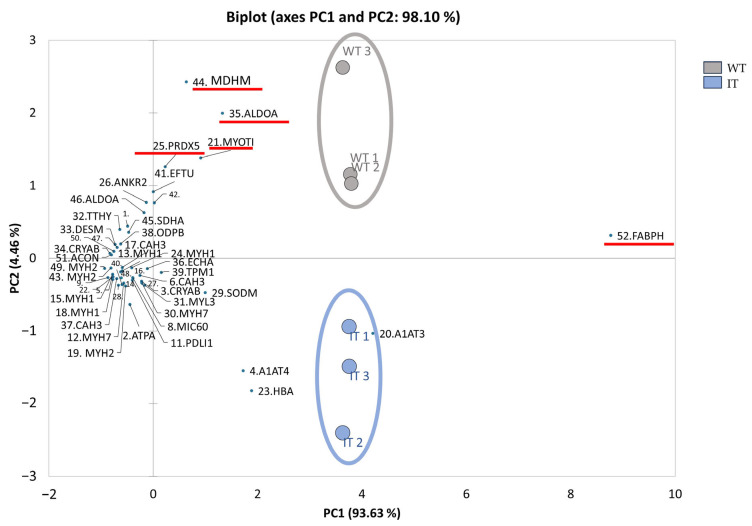
Supervised integrated Principal Component Analysis (PCA) and volcano plot analysis performed on the DASs. Red lines mark the most influential spots in the sample distributions. PCA summarizes 98.1% of the variance (PC1: 93.63% and PC2: 4.46%). Black numbers and acronyms indicate differential spots, whereas WT soleus samples are shown in gray and IT soleus samples in blue.

**Figure 3:**
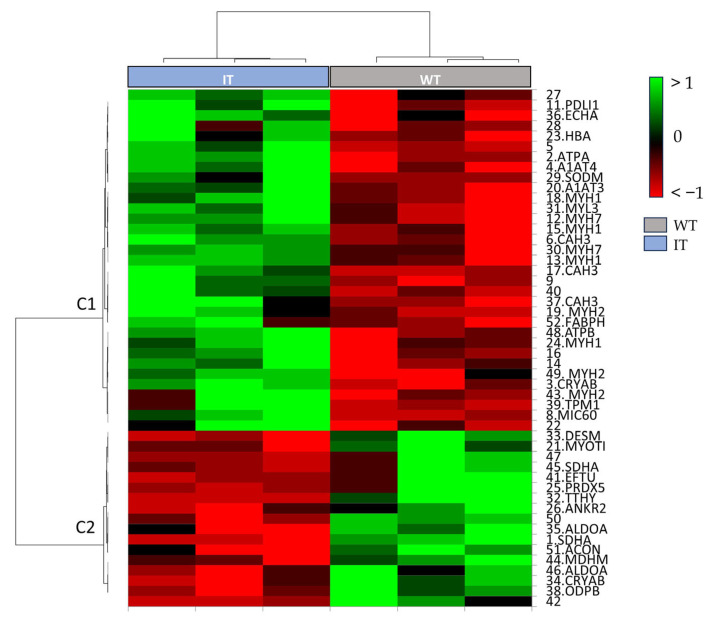
Heatmap analysis of the differential spots between WT vs. IT mouse soleus samples. Color changes from red to green indicate lower or higher protein abundance, respectively. Spot numbers and acronyms represent differential protein spots, while WT soleus samples are indicated in gray and IT soleus samples in blue. Unsuccessfully identified spots report only spot numbers.

We conducted a supervised multivariate analysis using the 50 differentially abundant spots (DASs). Principal Component Analysis (PCA) and heatmap analysis are shown in Figure 2 and Figure 3, respectively. Multivariate analysis shows that IT mice exhibit a distinct proteomic profile. In particular, the integrated PCA and volcano plot chart show a total variance of 98.06% and clearly separates the two conditions along the second main component (PC2). Based on the PCA, decreased abundance of peroxiredoxin-5 (PRDX5), myotilin (MYOTI), and fructose-bisphosphate aldolase A (ALDOA), and the increased abundance of fatty acid-binding protein (FABPH), circled in red, have greater relevance in sample stratification. The heatmap distinguished IT from WT conditions, clustering the DASs into the following two clusters (C1 and C2): C1 highlights the highly abundant proteins in the IT condition and includes proteins involved in structural and contractile functions, while C2 contains proteins involved in the regulation of metabolic processes.

### 2.2. Enrichment Analysis of the Proteomic Data

**Figure 4:**
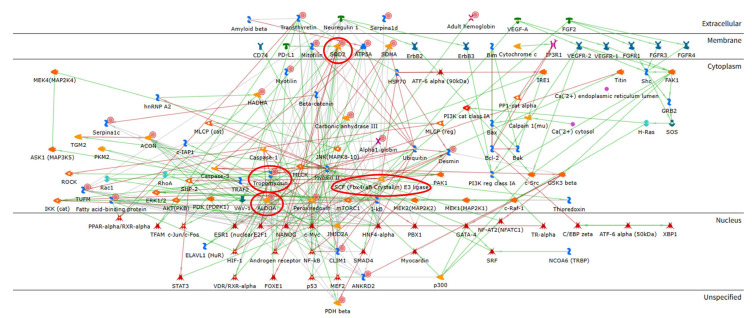
Network analysis performed using the MetaCore software (version 22.1 http://portal.genego.com, accessed on 23 April 2025, Clarivate Analytics, Philadelphia, PE, USA), to highlight characteristic protein interactions of differential abundant proteins. ALDOA, alpha-crystallin B, SOD2, and tropomyosin-1 are the central functional hubs of the net and are marked in red circles. (Symbol legend: https://portal.genego.com/legends/MetaCoreQuickReferenceGuide.pdf, accessed on 23 April 2025).

**Figure 5:**
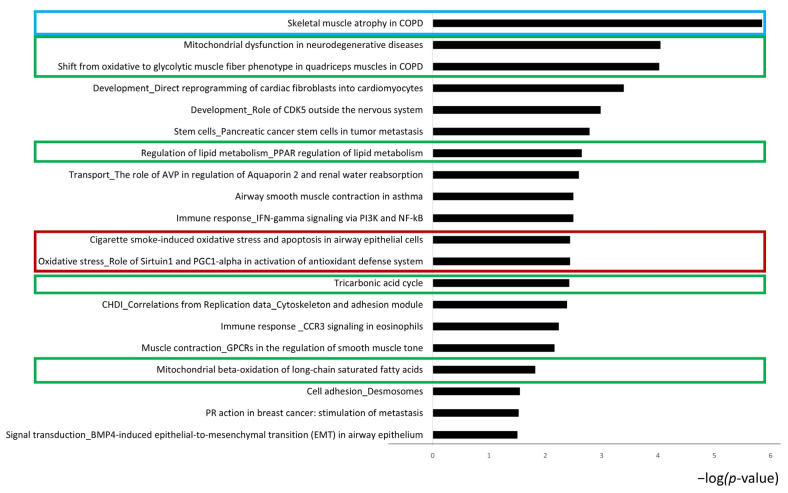
Histograms reporting the pathway maps involving DASs. Black histograms represent the −log *(p*-value) related to differential proteins resulting from the comparison between WT and IT mouse soleus samples. Particularly, the pathway maps analysis suggests terms related to the skeletal muscle structure (blue box), several terms related to metabolism, including metabolic shift (green boxes), and some terms suggesting an alteration of the oxidative stress response (red boxes).

To explore the molecular functions, signaling mechanisms, and networks of the identified proteins, an enrichment analysis was performed. Figure 4 shows the network analysis, highlighting the possible interactions that can occur among the differentially expressed proteins. ALDOA, alpha-crystallin B, SOD2, and tropomyosin-1 are the central functional hubs of the network (red circles). In Figure 5, the pathway maps analysis suggests terms related to the skeletal muscle structure (blue box), several terms related to metabolism (green boxes), and some terms suggesting an alteration in the oxidative stress response (red boxes). Therefore, the enrichment analysis suggested that CCD induces a complex alteration of the proteome, including structural and metabolic proteins.

### 2.3. Structural, Contractile, and Cytoskeletal Stabilization Proteins Were Altered

Proteomic analysis identified significant changes in proteins involved in muscle structure and contraction. In particular, we detected an abnormal increase in several fragments of different proteoforms of myosin (MYH1, MYH2, MYH7), which could be a direct consequence of hypotonia and generalized muscle weakness [26]. These data agree with previous structural characterization of IT mice, where Z-line streaming and sarcomere damage were typically found in type I fibers [21,27]. On the other hand, myotilin (MYOTI), which we found to be downregulated in IT, is a structural protein of the Z-disk that acts as stabilizer and anchors actin filaments at the Z-disk [28,29]. Decreased levels of this protein suggest an instability of actin filaments in muscle affected by CCD. Interestingly, two proteoforms of the alpha-crystallin B chain (CRYAB) were detected as differently abundant, indicating a change in its post-translational modification. CRYAB is a major chaperone in muscle [30] and modulates filament assembly and network formation. Indeed, as shown by the protein network in Figure 4, CRYAB binds to desmin (DESM) during its assembly into intermediate filaments. Their binding depends on the topology and surface characteristics of the DESM filament. This allows CRYAB to sense abnormal filament structures, influencing the structure and stability of these filaments and preventing improper protein folding and aggregation [31].

### 2.4. RYR1-I4898T Mutation May Induce Alteration in Metabolism

**Figure 6:**
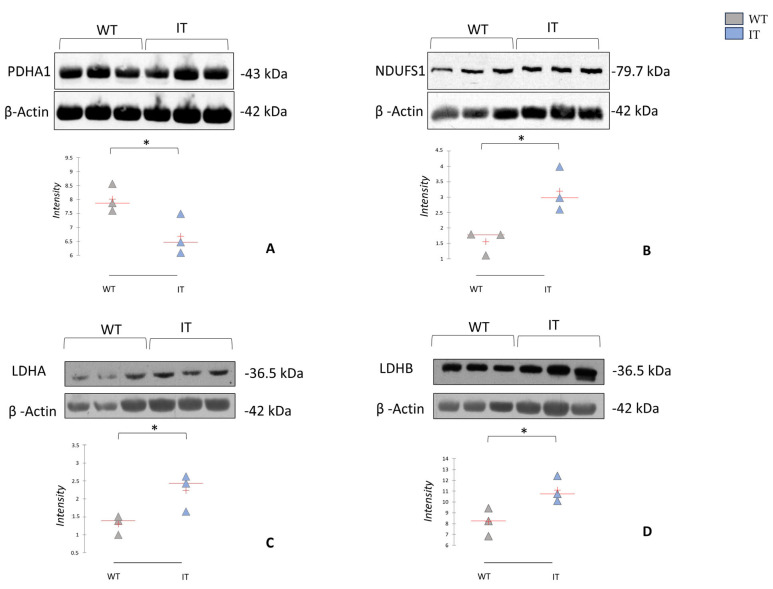
One-dimensional Western blot analysis with anti-PDHA1 (**A**), anti-NDUFS1 (**B**), anti-LDHA (**C**), and anti-LDHB (**D**) on soleus samples from WT and IT mice. Kruskal–Wallis statistical analysis with Dunn correction was performed. * indicate significant levels *p* ≤ 0.05.

The enrichment analysis by pathways maps performed on DASs also suggested several terms related to different metabolic processes, mitochondrial dysfunction, and stress response. Many of the altered metabolic proteins identified through differential proteomic analysis are involved in the respiratory chain and energy transport, glycolysis, the tricarboxylic acid cycle (TCA), and mitochondrial beta-oxidation of long-chain saturated fatty acids (Figure 5). A notable finding was the downregulation of proteins involved in glycolysis pathways, the pyruvate dehydrogenase complex, and the TCA cycle, particularly two proteoforms of fructose-bisphosphate aldolase A (ALDOA), the pyruvate dehydrogenase E1 component subunit beta (PDHB), two proteoforms of succinate dehydrogenase [ubiquinone] flavoprotein subunit (SDHA), malate dehydrogenase (MDHM), and aconitate hydratase (ACON), all with significant correlations (Figure S2). To further investigate the metabolic process highlighted by our proteomic analysis, we decided to test some key regulatory proteins, different from our DASs, using Western blot analysis on a different cohort of three WT and three IT mice. The results showed a significant decrease in pyruvate dehydrogenase alpha (PDHA) (Figure 6A), a component of the PDH complex that links glycolysis to the TCA cycle and represents a key step in the aerobic glycolytic process [32]. In light with these results, we hypothesized a probable shift from aerobic to anaerobic glycolysis. To test this, we performed Western blot analysis to assess the levels of lactate dehydrogenase A and B (LDHA and LDHB), which form an enzyme that converts pyruvate to lactate under hypoxic conditions, thereby allowing the regeneration of NAD+ from NADH and permitting the continuation of glycolysis [33]. In agreement with our hypothesis, we found a significant increase in the two LDH proteoforms in IT mice (Figure 6C,D). To the best of our knowledge, this aspect has not yet been investigated for CCD; however, it has already been seen in other pathologies associated with RYR1 mutations, such as malignant hyperthermia [5].

Conversely, several mitochondrial proteins involved in the respiratory and energy transport chain (e.g., trifunctional enzyme subunit alpha, creatine kinase M-type, and ATP synthase subunit α and β) were increased in IT mice, as highlighted by our proteomic analysis. To validate the involvement of the respiratory chain and energy transport process, we also performed Western blot to assess NDUFS1 (Figure 6B), which is the largest subunit of mitochondrial complex I and transfers electrons from NADH to the respiratory chain, using ubiquinone as the immediate electron acceptor [34]. The increased NDUFS1 levels in IT mice could confirm the enhanced oxidative phosphorylation in the CCD muscle [35]. Interestingly, Mic60 was increased in IT, this protein, a central component of the mitochondrial contact site and cristae organizing system (MICOS), is essential for maintaining mitochondrial membrane architecture, dynamics, and transcription of oxidative phosphorylation proteins [36].

**Figure 7:**
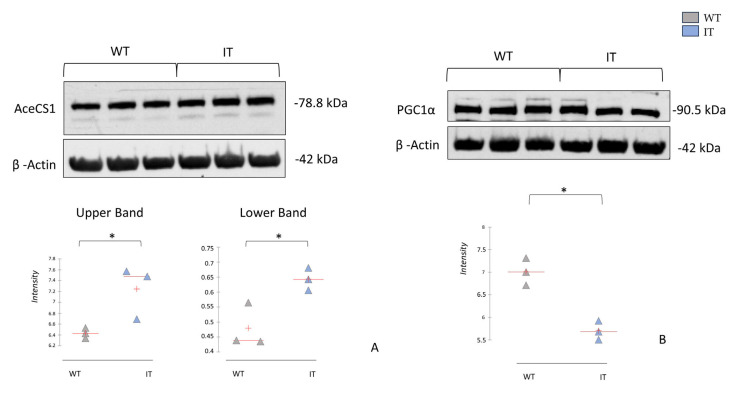
One-dimensional Western blot analysis with anti-AceCS1 (**A**) and anti-PGC1α (**B**) on soleus samples from WT and IT mice. Kruskal–Wallis statistical analysis with Dunn correction was performed. * indicate significant levels *p* ≤ 0.05.

Furthermore, as suggested by proteomic and enrichment analyses, we found an increase in proteins involved in fatty acid beta-oxidation (e.g., fatty acid-binding protein). To validate the potential increase in beta-oxidation metabolism, we tested the levels of acetyl-CoA synthetase (AceCS1), which catalyzes the conversion of acetate and CoA to acetyl-CoA, a key step in metabolic pathways such as fatty acid metabolism, the TCA cycle, and oxidative phosphorylation [37,38]. Interestingly, we found two proteoforms of this protein, both increased in the muscles of IT mice (Figure 7A). The increase in this enzyme may suggest an attempt by the CCD muscle to maximize its energy production capacity. However, pathway maps analysis also indicates the involvement of PGC1α (peroxisome proliferator-activated receptor gamma coactivator 1-alpha) (Figure 5, red square), which is strongly regulated by calcium signaling [39]. Given the pivotal role of this protein, we assessed its abundance by Western blot, highlighting its downregulation in IT mice (Figure 7B). PGC1α is a well-known transcriptional coactivator crucial for skeletal muscle metabolism [40]. Previous research highlighted the involvement of PGC-1α in regulating muscle mass and protein turnover, and a decreased level of PGC-1α has been associated with a greater degree of atrophy [41,42]. In general, a reduction in PGC1α levels may be attributed to an imbalance of calcium flux resulting from the RYR1 gene mutation. Our results are consistent with those reported by Lee et al., 2017 [20], who found that low levels of PGC1α in IT mice may be associated with increased mitochondrial damage. Given its central role in regulating the structure and metabolism of muscle fibers, PGC1α could be considered for further studies as a potential biomarker of target therapy in CCD.

### 2.5. RYR1-I4898T Mutation May Induce an Alteration in Oxidative Stress Response

Oxidative stress is a pathophysiological mechanism recurring in CCD and other RYR1-related myopathies [15,43]. Indeed, our proteomic analysis reveals alterations in the abundance of many proteins involved in the oxidative stress response, such as PDZ and LIM domain protein, superoxide dismutase, MICOS complex subunit Mic60, elongation factor Tu, peroxiredoxin-5, and ankyrin repeat domain-containing protein 2. A state of oxidative stress, such as that recognized in CCD, may lead to a metabolic shift to anaerobic glycolysis. Indeed, a slight increase in IIb/x fibers was reported in IT mice compared to controls [20]. Moreover, it was also reported that the production of lactate, which we hypothesize based on the upregulation of LDH, can in turn increase oxidative stress [44]. Our results are in agreement with the findings reported by Lee et al., 2017 [20], who observed an increase in oxidized proteins in IT mice muscles, which was associated with increased ER stress and ROS production in FDB fibers in IT mice compared to controls. Lee et al. explained that persistent increases in mitochondrial Ca^2+^ uptake and ROS production could lead to mitochondrial damage. Indeed, they observed a decrease in mitochondrial protein content, along with an increase in cytosolic cytochrome c levels and an increase in apoptotic nuclei and elevated levels of cleaved caspases 3, 9, and 12, as well as significant increase in caspase-3 activity and the expression of the p53 pro-apoptotic protein. Among the altered proteins, we also found several proteoforms of carbonic anhydrase 3 (CAH3), which were upregulated in IT mice. CAH3, a metalloenzyme, regulates pH and catalyzes the reversible hydration of carbon dioxide (CO_2_) to bicarbonate (HCO_3_) and a proton (H+) for the CO_2_ transport into the blood. CAH3 also protects from free radical damage [45,46]. Given that an increase in CAH3 is an established biomarker for muscle tissue damage [47,48], it could be considered as a biomarker for monitoring disease progression and therapeutic impact [49].

## 3. Materials and Methods

### 3.1. Animals

All animal-related procedures were performed with the highest standard of care to minimize animal discomfort and suffering. Mice were anesthetized with isoflurane and humanely euthanized by cervical dislocation, in accordance with the protocols approved by the Animal Care Committee of the University of Siena and the Italian Ministry of Health (64/2020-PR). All experimental procedures complied with Directive 2010/63/EU of the European Parliament and the Council of 22 September 2010 on the protection of animals used for scientific purposes. Experiments were conducted on adult (4 months old) C57Bl/6J mice, including both wild-type (WT) and heterozygous knock-in for *Ryr1*^I4895T^ (IT) mice. Animals had ad libitum access to food and water and were housed under controlled environmental conditions, with ambient temperature maintained between 21 and 25 °C, relative humidity at 50–60%, and a 12 h light/dark cycle. Soleus muscle samples were collected and immediately frozen at −80 °C until use.

### 3.2. Sample Preparation for Two-Dimensional Electrophoresis

Soleus sample preparation was performed as previously described [25]. Briefly, 10 mg of soleus muscle samples from three WT and three IT mice were divided into two sections using a scalpel and processed by two different extraction procedures, based on two different denaturing buffers. In particular, the protein extraction protocol using denaturation buffer A containing 2% *w*/*v* SDS and 1% *w*/*v* DTE (pH 6) and acetone precipitation provided a better extraction of high-MW proteins and sarcomere proteins. While denaturation buffer B, containing 8 M UREA, 4% *w*/*v* CHAPS, 1% *w*/*v* DTE, and 40 mM TRIS (pH 10) provided a better extraction of low-MW protein species, protein fragments, and mitochondria membrane proteins. Since the two procedures showed different patterns of extracted proteins, we performed two parallel differential proteomic analyses comparing the proteomic profile of the soleus tissues from WT and IT mice in order to acquire a thorough overview of the proteomic alterations induced by the pathology. Muscle samples were incubated with 40 µL of denaturation buffer A or B and homogenized using beads in a TissueLyser II (#85300 Qiagen, Venlo, PL, USA) at 2.5Hz for four 30 s cycle, with 1 min intervals. After bead removal, samples were centrifuged at 20,800× *g* for 15 min at 4 °C, and the supernatants were recovered. Protein concentrations were estimated by the Bradford protocol [50].

### 3.3. Two-Dimensional Electrophoresis

Protein extracts were resolved by two-dimensional electrophoresis (2DE) using the Immobiline–polyacrylamide system, as described by Vantaggiato et al. [51]. For analytical gels, 60 μg of protein were mixed with 0.2% (*v*/*v*) carrier ampholyte, whereas for protein identification, 600 µg of protein were combined with 2% (*v*/*v*) carrier ampholyte. Samples were diluted in 350 μL of lysis buffer (8 M UREA, 4% *w*/*v* CHAPS, 1% *w*/*v* DTE) with traces of bromophenol blue, and loaded onto 18 cm nonlinear pH 3-10 IPG strips (Cytiva, Marlborough, MA, USA) for isoelectric focusing (IEF). IEF was performed at 16 °C using the Ettan™ IPGphor™ Manifold system (Cytiva, Marlborough, MA, USA), under the following voltage program: 0 V for 1 h, 30 V for 7 h, 200 V for 1 h, 300 V for 30 min, a gradient until 3500 V in 2 h, 3500 V for 10 min, from 3500 V to 5000 V in 30 min, 5000 V for 30 min, from 5000 V to 8000 V in 1 h, and 8000 V for the rest of the run until reaching a total of 95,000 VhT. After focusing, strips were equilibrated in two steps: the first for 12 min in 6 M UREA, 2% *w*/*v* SDS, 2% *w*/*v* DTE, 30% *v*/*v* glycerol, and 0.05 M TRIS-HCL (pH 6.8), then for 5 min in 6 M UREA, 2% *w*/*v* SDS, 2.5% *w*/*v* iodoacetamide, 30% *v*/*v* glycerol, 0.05 M TRIS-HCL (pH 6.8), and a trace of bromophenol blue. Second-dimensional separation was performed on 9–16% SDS gradient polyacrylamide gels (18 cm × 20 cm × 1.5 mm) at 40 mA/gel and 9 °C until the dye front reached the bottom of the gel. Analytical gels were stained using ammoniacal silver staining [52], while MS-preparative gels were stained following an MS-compatible silver staining protocol [53]. All gels were digitalized using an Image Scanner III laser densitometer provided with LabScan 6.0 software (Cytiva).

### 3.4. Image and Statistical Analysis

Two-dimensional gel images were processed using the Melanie 9.0 software (Swiss Institute of Bioinformatics, Quartier Sorge, Batiment Amphipole 1015 Lausanne, Switzerland). Gels from the same condition were aligned to a high-resolution master reference gel, and inter-class comparison were subsequently performed. Statistical analysis was carried out using Melanie 9.0 and XLStat version 2021.2.2, which performed, respectively, an ANOVA test and Benjamini–Hochberg FDR on the percentage of relative volume (%V) (integration of optical density of a single spot (volume) divided by the total volume of spots and expressed as a percentage). Protein spots were considered differentially abundant when the fold-change in %V was ≥1.5-fold and *p*-value < 0.05. Supervised Principal Component Analysis (PCA) and heatmap analysis were performed by XLStat. Pearson’s correlation tests were applied to assess linear correlation relationships among DASs.

### 3.5. MALDI-TOF Identification by PMF

Differential protein spots were manually excised from gels and destained using a solution of 30 mM potassium ferricyanure and 100 mM sodium tiosulphate anhydrous, followed by incubation in 200 mM ammonium bicarbonate. Spots were dehydrated in 100% acetonitrile (ACN), rehydrated with trypsin solution, and digested overnight at 37 °C. Peptide extracts (0.75 µL) were spotted on an MALDI target, air-dried, and overlaid with 1 µL of matrix solution containing 5 mg/mL α-cyano-4-hydroxycinnamic acid (CHCA) dissolved in 50% *v*/*v* ACN and 5% *v*/*v* trifluoroacetic acid (TFA). Mass spectra were acquired using an UltrafleXtreme™ MALDI-ToF/ToF instrument (Bruker Corporation, Billerica, MA, USA), according to Landi C et al. [54]. Spectra were processed with FlexControl software version 3.0 (Bruker) and Flex Analysis software version 3.0 (Bruker). Trypsin autolysis peaks were used for internal calibration, and mass lists were filtered to remove contaminants such as keratin- and matrix-related ions. Peptide Mass Fingerprinting (PMF) searches were performed using the MASCOT (Matrix Science Ltd., London, UK, http://www.matrixscience.com, accessed on 23 April 2025) search engine with the following parameters: Swiss-Prot/TrEMBL and NCBInr as databases, *Mus musclulus* as taxonomy, 20 ppm mass tolerance, one missed cleavage, carbamidomethylation of cysteine as a fixed modification, and oxidation of methionine as a variable modification. Proteomic data were deposited in the ProteomeXchange Consortium via PRIDE [55] under dataset identifier PXD069980.

### 3.6. MetaCore Enrichment Analysis

Identified proteins were analyzed using MetaCore version 22.1 build (http://portal.genego.com, accessed on 23 April 2025, Clarivate Analytics, Philadelphia, PA, USA). The platform integrates curated database of protein–protein and protein–DNA interactions, transcription factors, and signaling pathways. Pathway enrichment was evaluated using canonical maps ranked by statistical significance (*p* ≤ 0.001) based on Gene Ontology annotations. Additionally, a shortest-path algorithm was applied to conduct interaction network analyses linking experimental proteins with MetaCore database entities.

### 3.7. Western Blot Analysis

Western blotting was performed on soleus muscles samples from an independent cohort of WT and IT mice (n = 3 per group). Protein extracts (40 μg) were diluted in Leamly buffer (2% *w/v* SDS, 100 mM Tris–HCl pH 6.8, 4% *v/v* β-mercaptoethanol, 20% *v/v* glycerol), boiled at 95 °C for 5 min, separated on 12% SDS-PAGE gels, and transferred onto nitrocellulose membranes (Cytiva). Membranes were blocked (3% skimmed milk, 0.1% Triton X-100 in PBS, pH 7.4), incubated overnight at 4 °C with primary antibodies, and then for 2 h with goat peroxidase-conjugated anti-rabbit immunoglobulin G (Sigma, St. Louis, MO, USA, working dilution 1:7000). After three washing steps in blotting solution, one washing step in Triton X100 0.5% *v/v* in PBS pH 7,4 and other two washing steps in 0.05 M Tris-HCl pH 6.8, bands were visualized using ECL chemiluminescence detection system (Cytiva) and exposing membranes to Hiyperfilm ECL X-ray films (Cytiva). Densitometric analysis was performed using the ImageMaster 2D Platinum v. 6.0 software. Immunoblot data were then exported into Excel (Microsoft Office) to perform Kruskal–Wallis two-tailed test statistical analysis and the post hoc Dunn’s test for multiple comparisons using XLStat version 2021.2.2. Samples were normalized to β-actin.

### 3.8. Antibodies

The primary antibodies used in this study were as follows: Anti-pyruvate dehydrogenase (PDH) (Cell Signaling, Danvers, MA, USA, Cat. #3205, WB 1:1000); Anti-LDHA (Cell Signaling, Cat. #3582, WB 1:1000); Anti-LDHB (Cell Signaling, Cat. #56298, WB 1:1000); Anti-AceCS1 (Cell Signaling, Cat. #3658, WB 1:1000); Anti-PGC1 alpha Antibody (Antibodies, Cat. #A8783, WB 1:1000); Anti-NDUFS1 (Cell Signaling, Cat. # 70264, WB 1:1000); according to the manufacturer’s instructions.

## 4. Conclusions

In conclusion, our proteomic analysis revealed alterations in muscle structural and contraction proteins, likely associated with pathology. In particular, the high presence of myosin fragments, the altered abundance of alpha-crystallin B chain proteoforms, and a decrease in desmin and myotilin suggest a condition of general structural instability in CCD-affected muscle. Furthermore, the data obtained from our study revealed a reduction in glycolytic and TCA cycle activity, accompanied by an increase in lactate dehydrogenase and proteins involved in fatty acid beta-oxidation and oxidative phosphorylation, as also proposed by bioinformatics analysis. This suggests a metabolic shift from aerobic to anaerobic glycolysis in CCD muscle and an attempt at lipid catabolism to maximize energy production. This would also lead to an increase in oxidative stress and the corresponding alteration of stress response proteins. Finally, future analysis could expand the number of samples and validate the lower levels of PGC1α in individuals affected by CCD.

## Figures and Tables

**Table 1:** Protein identification by mass spectrometry, showing spot numbers corresponding to Figure 1 and Figure S1, protein names, UniProt names, accession numbers, and gene names. The table also reports the following statistical results: ANOVA *p*–value, adjusted *p*–value (Benjamini–Hochberg FDR), the V% mean for each condition, and fold-change. The most abundant values are marked in bold. The theoretical pI and MW of identified proteins and MASCOT search results (score, matched peptides, coverage%). Unsuccessfully identified spots report only proteomic and statistical data.

Spot N.	Protein Name	Entry Name	Gene Name	AC	ANOVA Test				FC	Theoretical pI–MW	MASCOT Search Results		
					Anova (*p*)	Adjusted (*p*) (FDR)	WT	IT			Score	No. of Matched Peptides	Sequence Coverage (%)
1	Succinate dehydrogenase [ubiquinone] flavoprotein subunit. mitochondrial	SDHA_MOUSE	Sdha	Q8K2B3	0.0002	0.011	**0.0228**	0.0113	2.01	7.06–73,623	150	12/16	18
2	ATP synthase subunit alpha. mitochondrial Fragment C	ATPA_MOUSE	Atp5f1a	Q03265	0.002	0.0361	0.0096	**0.0349**	3.64	9.22–59,830	65	6/10	14
3	Alpha-crystallin B chain	CRYAB_MOUSE	Cryab	P23927	0.0035	0.0361	0.0191	**0.0398**	2.09	6.76–20,056	189	12/18	62
4	Alpha-1-antitrypsin 1–4	A1AT4_MOUSE	Serpina1d	Q00897	0.0042	0.0361	0.0619	**0.1419**	2.29	5.24–46,140	125	8/12	24
5					0.0045	0.0361	0.0051	**0.0124**	2.43				
6	Carbonic anhydrase 3	CAH3_MOUSE	Ca3	P16015	0.0073	0.0387	0.0214	**0.0342**	1.6	6.89–29,633	204	12/17	69
8	MICOS complex subunit Mic60	MIC60_MOUSE	Immt	Q8CAQ8	0.0097	0.0387	0.0162	**0.0299**	1.85	6.18–84,247	98	7/8	15
9					0.0102	0.0387	0.0027	**0.0098**	3.68				
11	PDZ and LIM domain protein 1	PDLI1_MOUSE	Pdlim1	O70400	0.0136	0.0387	0.0157	**0.0299**	1.9	6.38–36,208	101	9/17	34
12	Myosin-7 Fragment C	MYH7_MOUSE	Myh7	Q91Z83	0.0154	0.0387	0.0114	**0.0227**	2	5.59–223,539	124	23/38	13
13	Myosin-1 Fragment C	MYH1_MOUSE	Myh1	Q5SX40	0.0162	0.0387	0.0113	**0.0176**	1.55	5.60–224,116	148	21/29	11
14	Actin. alpha skeletal muscle	ACTS_MOUSE	Acta1	P68134	0.0179	0.0387	0.0095	**0.0207**	2.18	5.23–42,366	221	17/29	58
15	Myosin-1 Fragment C	MYH1_MOUSE	Myh1	Q5SX40	0.0191	0.0387	0.0065	**0.0116**	1.78	5.60–224,116	72	13/22	7
16	Actin. alpha skeletal muscle	ACTS_MOUSE	Acta1	P68134	0.0192	0.0387	0.0163	**0.0299**	1.83	5.23–42,366	75	6/14	19
17	Carbonic anhydrase 3	CAH3_MOUSE	Ca3	P16015	0.0213	0.0401	0.0121	**0.019**	1.57	6.89–29,633	189	11/15	61
18	Myosin-1 Fragment C	MYH1_MOUSE	Myh1	Q5SX40	0.0276	0.047	0.0059	**0.0131**	2.21	5.60–224,116	210	26/32	14
19	Myosin-2	MYH2_MOUSE	Myh2	G3UW82	0.0324	0.049	0.0104	**0.0274**	2.63	5.61–224,050	105	14/19	10
20	Alpha-1-antitrypsin 1–3	A1AT3_MOUSE	Serpina1c	Q00896	0.0359	0.049	0.145	**0.2292**	1.58	5.25–45,966	202	14/18	40
21	Myotilin	MYOTI_MOUSE	Myot	Q9JIF9	0.0372	0.049	**0.0765**	0.0506	1.51	9.02–55,738	204	16/22	36
22					0.0382	0.049	0.006	**0.0131**	2.2				
23	Hemoglobin subunit alpha	HBA_MOUSE	Hba	P01942	0.0383	0.049	0.0651	**0.151**	2.32	7.96–15,133	104	7/12	39
24	Myosin-1 Fragment C	MYH1_MOUSE	Myh1	Q5SX40	0.0384	0.049	0.017	**0.0267**	1.56	5.60–224,116	106	14/17	8
25	Peroxiredoxin-5. mitochondrial	PRDX5_MOUSE	Prdx5	P99029	0.0392	0.049	**0.0526**	0.0268	1.96	9.10–22226	116	6/7	40
26	Ankyrin repeat domain–containing protein 2	ANKR2_MOUSE	Ankrd2	Q9WV06	0.0415	0.0496	**0.0363**	0.0208	1.75	5.95–36,855	273	18/23	67
27	Actin. alpha skeletal muscle	ACTS_MOUSE	Acta1	P68134	0.0444	0.0496	0.0196	**0.0388**	1.98	5.23–42,366	139	12/23	35
28	Actin. alpha skeletal muscle	ACTS_MOUSE	Acta1	P68134	0.0473	0.0497	0.0073	**0.0195**	2.66	5.23–42,366	109	9/15	28
29	Superoxide dismutase [Mn]. mitochondrial	SODM_MOUSE	Sod2	P09671	0.0497	0.0497	0.0543	**0.0901**	1.66	5.57–33,118	94	7/19	37
30	Myosin-7 Fragment C	MYH7_MOUSE	Myh7	Q91Z83	0.0497	0.0497	0.0223	**0.0367**	1.65	5.59–223,539	199	25/29	14
31	Myosin light chain 3	MYL3_MOUSE	Myl3	P09542	0.0072	0.0387	0.0224	**0.0403**	1.8	5.03–22,521	154	10/14	51
32	Transthyretin	TTHY_MOUSE	Ttr	P07309	0.0087	0.0387	**0.0179**	0.0058	3.07	5.77–15,880	96	5/8	43
33	Desmin	DESM_MOUSE	Des	P31001	0.0134	0.0387	**0.0131**	0.0057	2.29	5.21–53,465	180	13/16	32
34	Alpha–crystallin B chain	CRYAB_MOUSE	Cryab	P23927	0.0139	0.0387	**0.0085**	0.004	2.14	6.76–20,056	120	8/13	49
35	Fructose–bisphosphate aldolase A	ALDOA_MOUSE	Aldoa	P05064	0.0173	0.0387	**0.0945**	0.0576	1.64	8.31–39787	120	9/11	30
36	Trifunctional enzyme subunit alpha. mitochondrial	ECHA_MOUSE	Hadha	Q8BMS1	0.0168	0.0387	0.026	**0.039**	1.5	9.24–83,302	101	7/7	9
37	Carbonic anhydrase 3	CAH3_MOUSE	Ca3	P16015	0.0194	0.0387	0.0074	**0.0172**	2.33	6.89–29,633	255	14/17	70
38	Pyruvate dehydrogenase E1 component subunit beta. mitochondrial	ODPB_MOUSE	Pdhb	Q9D051	0.0216	0.0401	**0.0163**	0.0099	1.65	6.41–39,254	56	5/9	16
39	Tropomyosin alpha-1 chain	TPM1_MOUSE	Tpm1	P58771	0.028	0.047	0.0328	**0.0516**	1.57	4.69–32,718	112	9/14	19
40					0.0327	0.049	0.0092	**0.0233**	2.53				
41	Elongation factor Tu. mitochondrial	EFTU_MOUSE	Tufm	Q8BFR5	0.0366	0.049	**0.0421**	0.0234	1.8	7.23–49,876	236	17/21	37
42					0.0396	0.049	**0.0445**	0.0231	1.92				
43	Myosin-2 Fragment C	MYH2_MOUSE	Myh2	G3UW82	0.0386	0.049	0.0062	**0.0093**	1.5	5.61–224,050	82	9/12	6
44	Malate dehydrogenase. mitochondrial	MDHM_MOUSE	Mdh2	P08249	0.042	0.0496	**0.0756**	0.0265	2.86	8.93–36,053	104	7/12	30
45	Succinate dehydrogenase [ubiquinone] flavoprotein subunit. mitochondrial	SDHA_MOUSE	Sdha	Q8K2B3	0.0429	0.0496	**0.0224**	0.0136	1.64	7.06–73,623	114	8/9	14
46	Fructose-bisphosphate aldolase A	ALDOA_MOUSE	Aldoa	P05064	0.0457	0.0497	**0.035**	0.0196	1.78	8.31–39,787	155	8/10	24
47					0.0461	0.0497	**0.0134**	0.0085	1.57				
48	ATP synthase F(1) complex catalytic subunit beta. mitochondrial	ATPB_MOUSE	Atp5f1b	P56480	0.0049	0.0361	0.0115	**0.0199**	1.73	5.19–56,265	210	14/17	39
49	Myosin–2 Fragment C	MYH2_MOUSE	Myh2	G3UW82	0.0139	0.0387	0.0026	**0.0043**	1.64	5.61– 224050	113	14/17	8
50					0.0032	0.0361	**0.011**	0.0067	1.64				
51	Aconitate hydratase. mitochondrial Fragment C	ACON_MOUSE	Aco2	Q99KI0	0.0274	0.047	**0.009**	0.0058	1.54	8.08–86151	145	12/17	21
52	Fatty acid-binding protein. heart	FABPH_MOUSE	Fabp3	P11404	0.0494	0.0497	0.2797	**0.4205**	1.5	6.11–14810	183	12/23	73

## Data Availability

The mass spectrometry proteomics data have been deposited in the ProteomeXchange Consortium via the PRIDE partner repository. Project name: Molecular Insights into Central Core Disease: Proteomic Signatures and Potential Therapeutic Biomarkers in RYR1 I4895T Mice. Project accession: PXD069980. Access details: Log in to the PRIDE website using the following details: Project accession: PXD069980; Token: 43uJ3BIJiENf.

## Supplementary Materials

The following supporting information can be downloaded at: https://www.mdpi.com/article/10.3390/ijms262311451/s1.
