# Supplementary material for: Molecular Insights into Central Core Disease: Proteomic Signatures and Potential Therapeutic Biomarkers in RYR1 I4895T Mice
Source: Int J Mol Sci. 2025 Nov 26;26(23):11451. doi: 10.3390/ijms262311451 (PMC12691891; doi:10.3390/ijms262311451)
Supplement: Supplementary file 1 [file ijms-26-11451-s001.zip › ijms-3982859-supplementary.pdf]

Extraction With denaturation buffer A 2% SDS and 1% DTE

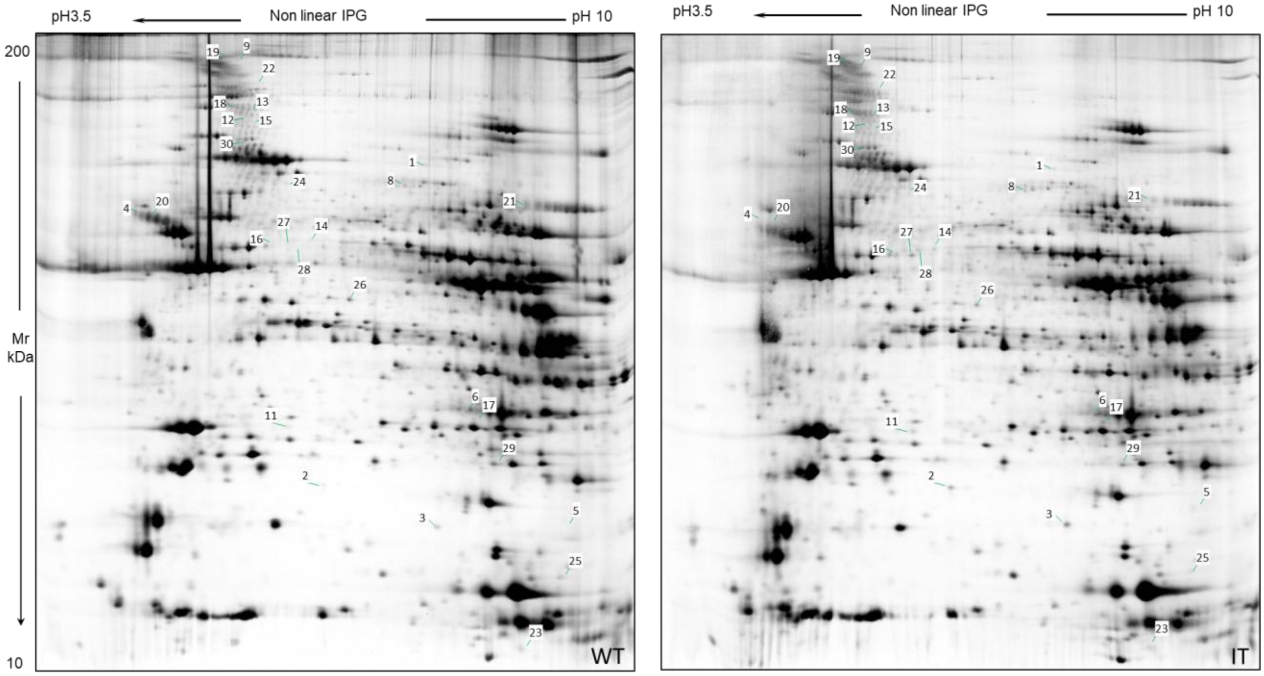

Extraction With denaturation buffer B 8 M UREA, 4% CHAPS, 1% DTE and 40 mM TRIS

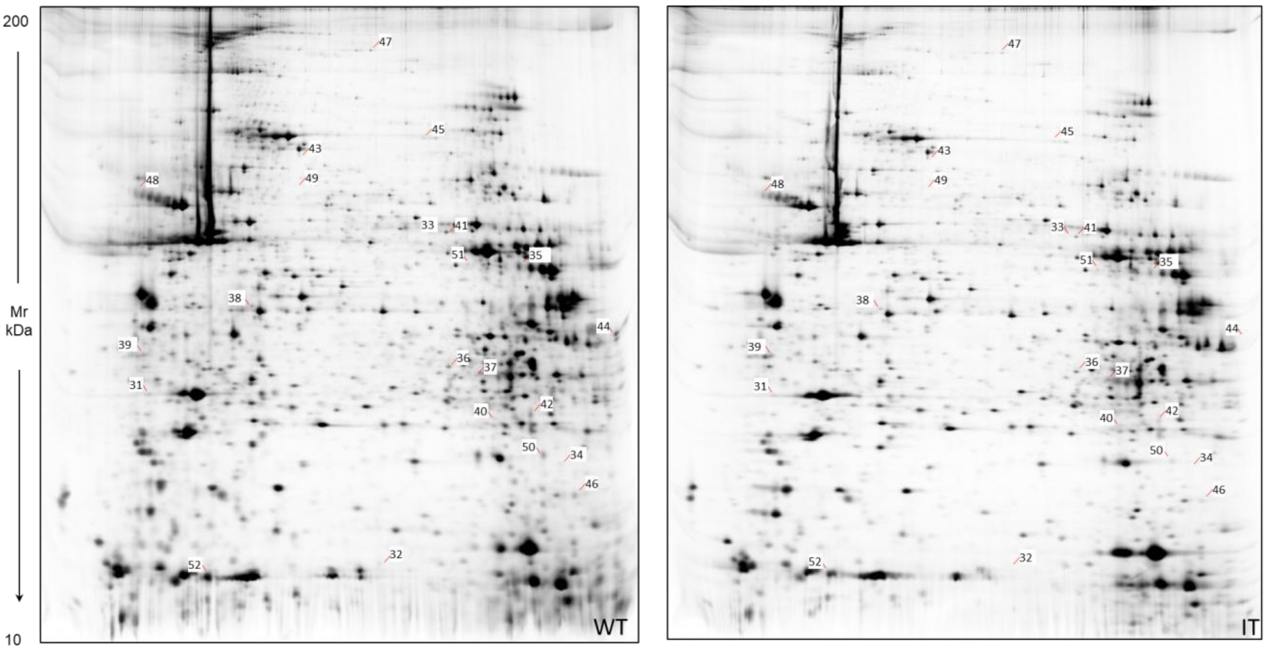

Figure S1. Reference gel maps from two different extraction method. WT and IT soleus reference gel maps reporting the statistically relevant spots ($p \leq 0.05$) with a fold change ratio ≥ 1.5, obtained from two different extraction method.

Pearson's correlation test

Variables	35.ALDOA	38.ODPB	46.ALDOA
35.ALDOA	**1**	**0.868**	**0.870**
38.PDHB	**0.868**	**1**	**0.966**
46.ALDOA	**0.870**	**0.966**	**1**

A

Variables	44.MDHM	1.SDHA	45.SDHA	51.ACON
44.MDHM	**1**	**0.903**	**0.818**	**0.867**
1.SDHA	**0.903**	**1**	**0.858**	**0.891**
45.SDHA	**0.818**	**0.858**	**1**	**0.878**
51.ACON	**0.867**	**0.891**	**0.878**	**1**

B

significance level p<0,05

Figure S2. Pearson's correlation test . A. Pearson's correlation between the %V of PDHB and ALDOA (spot 35, 38 and 46) in soleus samples. Values are shown with Pearson's correlation coefficients (r). B. Pearson's correlation between the %V of SDHA, MDHM and ACON (spot 45 and 1, 44, 51) in soleus samples. Values are shown with Pearson's correlation coefficients (r).
